# Crosstalk between Ethylene, Jasmonate and ABA in Response to Salt Stress during Germination and Early Plant Growth in *Cucurbita pepo*

**DOI:** 10.3390/ijms25168728

**Published:** 2024-08-10

**Authors:** Sonsoles Alonso, Keshav Gautam, Jessica Iglesias-Moya, Cecilia Martínez, Manuel Jamilena

**Affiliations:** Department of Biology and Geology, Agri-Food Campus of International Excellence (CeiA3) and Research Center CIAMBITAL, University of Almería, 04120 Almería, Spain; sad503@ual.es (S.A.); kkg480@ual.es (K.G.); yim100@ual.es (J.I.-M.)

**Keywords:** ethylene, ABA, jasmonate, crosstalk, germination, seedling, salt stress, *Cucurbita pepo*

## Abstract

The crosstalk of phytohormones in the regulation of growth and development and the response of plants to environmental stresses is a cutting-edge research topic, especially in crop species. In this paper, we study the role and crosstalk between abscisic acid (ABA), ethylene (ET), and jasmonate (JA) in the control of germination and seedling growth in water or in standard nutrient solution and under salt stress (supplemented with 100–200 mM NaCl). The roles of ET and JA were studied using squash ET- and JA-deficient mutants *aco1a* and *lox3a*, respectively, while the crosstalk between ET, JA, and ABA was determined by comparing the expression of the key ABA, JA, and ET genes in wild-type (WT) and mutant genotypes under standard conditions and salt stress. Data showed that ET and JA are positive regulators of squash germination, a function that was found to be mediated by downregulating the ABA biosynthesis and signaling pathways. Under salt stress, *aco1a* germinated earlier than WT, while *lox3a* showed the same germination rate as WT, indicating that ET, but not JA, restricts squash germination under unfavorable salinity conditions, a function that was also mediated by upregulation of ABA. ET and JA were found to be negative regulators of plant growth during seedling establishment, although ET inhibits both the aerial part and the root, while JA inhibits only the root. Both *aco1a* and *lox3a* mutant roots showed increased tolerance to salt stress, a phenotype that was found to be mainly mediated by JA, although we cannot exclude that it is also mediated by ABA.

## 1. Introduction

Phytohormones regulate the growth of the entire plant life cycle and are responsible for modulating its adaptive response to biotic and abiotic stresses [1,2]. Hormone homeostasis in seeds maintains the dormancy of the mature seed until the appropriate environmental time for germination. Abscisic acid (ABA) and gibberellins (GA) are the primary phytohormones that inhibit or promote germination, respectively, in response to environmental cues [3,4,5,6], with the ABA/GA ratio determining the progress or cessation of germination [7,8]. In recent years, other phytohormones have been found to be involved in germination, but their function is likely mediated by biosynthesis and signaling of the main phytohormones ABA and GA [9].

Ethylene (ET) stimulates the release of dormancy and promotes germination in several plant species [10,11,12,13], and its crosstalk with ABA has been reported to take place through ethylene receptors [14]. Auxin alone is not considered a key regulator of seed germination, but has been shown to interact with ABA during germination [15,16,17]. Mei et al. [18] have recently shown that exogenous auxin works synergistically with JA to enhance the ABA-induced delay of Arabidopsis germination. The role of jasmonate (JA) in germination remains unclear. JA has been proposed to act synergistically with ABA to delay seed germination in Arabidopsis, where ABA can promote JA biosynthesis through the SAPK10-bZIP72-AOC pathway, and JA activates ABA signaling through the physical interaction between JAZ and ABI3/ABI5 [9,19,20]. However, mutants in JA signaling genes like receptor CORONATINE INSENSITIVE1 (*COI1*), *JAZ*, and *MYC2* (a bHLH TF) exhibit no impaired germination [9]. Brassinosteroids (BRs) promote seed germination and have also been demonstrated to crosstalk with ABA through BRASSINOSTEROID INSENSITIVE 2, a key repressor of BR signaling, which phosphorylates and stabilizes ABI5 to mediate ABA signaling [21]. Cytokinins induce ABI5 degradation, thus limiting the effects of ABA and promoting germination [22,23]. Under normal conditions, salicylic acid (SA) inhibits germination by suppressing the expression of GA-induced α-amylase genes [24]. Therefore, the hormonal network controlling germination is much more complex than limiting the crosstalk of phytohormones with ABA or GA, and we are still far from fully understanding it.

Phytohormones also play important but contrasting roles in the response of plants to salt stress. Soil salinity is one of the main factors limiting the sustainable development of food and feed production [25]. An increasing amount of arable land is threatened by salinity due to improper irrigation and drainage, backflow of seawater, or the abuse of chemical fertilizers, among other reasons. Salinity conditions lead to inhibition of growth, reduction in crop yield, and even plant death [26]. ABA is known to be a positive regulator of the defensive response of plants, while the effect of ethylene on salt tolerance is species-specific. In Arabidopsis, tomato, and maize, ET positively regulates salinity tolerance [27,28,29], whereas in rice, tobacco, and zucchini, ET plays a negative role in the response to salt stress [26,30,31].

Similarly to ABA, salt stress has been observed to cause higher levels of JA in leaves and roots, and the induction of genes related to JA biosynthesis, which is clear evidence of the involvement of JA in the plant response to salt. Some reports showed that exogenous application of JA or methyl JA (MeJA) confers increased salt stress tolerance in pepper, pea, barley, wheat, and soybean [32,33,34,35,36], suggesting a positive role of JA in plant salt stress tolerance. However, other studies in rice and grapevine have reached the opposite conclusion [37,38,39]. Therefore, the role of JA in tolerance to plant salt stress remains unclear and controversial.

Investigating the crosstalk between phytohormones involved in the adaptation of plants to salt stress at different stages of plant development is currently a popular research area, especially in crops of economic importance. Recently, Cebrián et al. [40,41] reported two *Cucurbita pepo* mutants, *aco1a* and *lox3a*, that are deficient in ET and JA, respectively. These mutants can be very useful for deciphering the function of both ET and JA in different developmental and physiological processes. Since the function of these phytohormones in the germination and growth of plants under normal and saline conditions is completely unknown in squash, in this paper we study the response of *aco1a* and *lox3a* mutants to salt stress during both germination and seedling establishment and analyze the gene expression of key genes of the ABA, ET, and JA biosynthesis and signaling pathways. Physiological and molecular results revealed a crosstalk between ET, JA, and ABA in controlling germination, plant growth, and salt stress response in *C. pepo*. 

## 2. Results

### 2.1. Response of aco1a and lox3a to Salt Stress during Germination and Early Radicle Elongation

**Figure 1:**
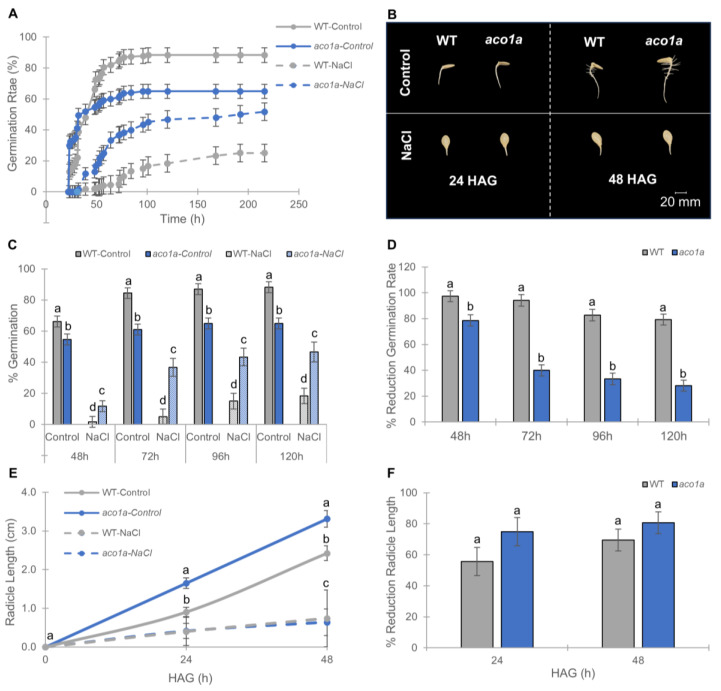
Response of *aco1a* to salt stress during germination and early radicle growth in *C. pepo.* (**A**) Germination rate of WT and mutant *aco1a* mutant seeds under control (solid lines) and saline conditions (dashed lines). Seeds were imbibed for 16 h in distilled water (control) and 200 mM NaCl and then allowed to germinate between two filter papers soaked in the same solution in Petri dishes. The percentage of germination was assessed every 2 h up to 216 h. (**B**) WT and *aco1a* seeds at different hours after germination (HAG) under control and salt conditions. (**C**) Percentage of germination of WT and *aco1a* seeds at 48, 72, 96, and 120 h after imbibition in water and 200 mM NaCl. (**D**) Percentages of reduction in germination in response to salt stress in WT and *aco1a* seeds with respect to those of the same genotype germinated in water. (**E**) Effect of *aco1a* mutation and salt stress on radicle length at 24 and 48 HAG. (**F**) Percentages of reduction in radicular length in response to salt stress in WT and *aco1a* seeds compared to those of the same genotype growing in water. Data are mean ± SE (n = 70). The error bars represent SE. Different letters indicate statistically significant differences (*p* ≤ 0.05) between samples taken at the same time.

To gain insight into the role of ethylene and JA in the germination of *C. pepo,* the germination rate of the *aco1a* and *lox3a* seeds was compared with that of the *wild type* (WT) under both water (control) and salt stress (200 mM NaCl) conditions. Figure 1 shows the germination rates of the ethylene-deficient mutant *aco1a* compared to that of the WT for 216 h. In water, the germination rate of *aco1a* was lower than that of the WT (65% in *aco1a* versus 88.3% in the WT 120 h after imbibition), indicating that ET produced by *CpACO1A* improves *C. pepo* germination. Salt treatment reduced the germination rate of both the WT and the mutant, but the mutant was less affected by NaCl than the WT (Figure 1A–C). The percentages of germination at 48, 72, 96, and 120 h of *aco1a* seeds under salt stress were always significantly higher than those of WT (Figure 1C). Furthermore, the percentages of reduction in the germination rate were significantly lower in the mutant than in the WT at all analyzed time points (Figure 1D), indicating that *aco1a* is more tolerant to salt stress than the WT during germination. The germination of the mutant was, in fact, less affected by salt than the germination of the WT. 

Radicle length was assessed 24 and 48 h after germination (HAG). The *aco1a* radicle was differentially larger than that of the WT under control conditions, indicating an inhibitory effect of ET on the radicle elongation (Figure 1E). However, the length of the radicle was similarly reduced by salt stress in both the WT and mutant (Figure 1F).

**Figure 2:**
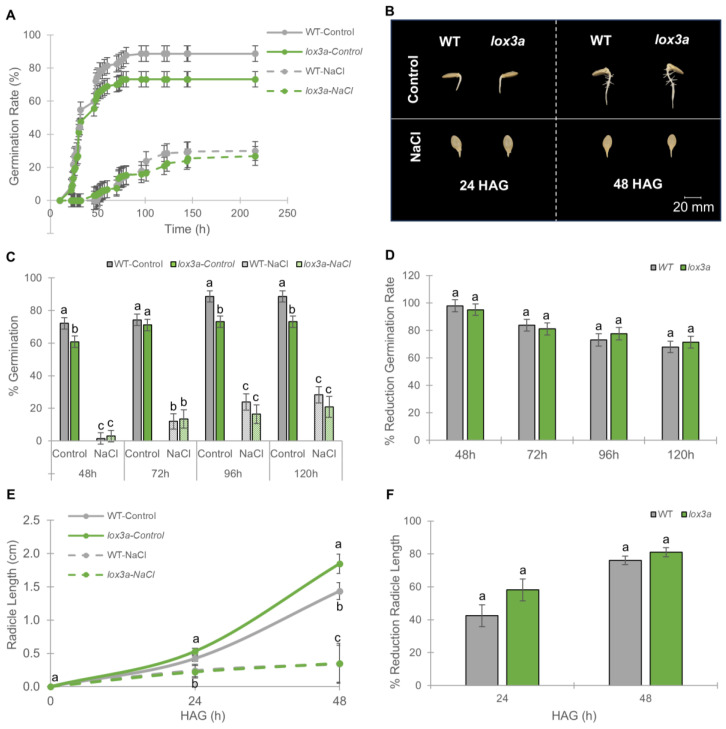
Response of *lox3a* to salt stress on germination and early radicle growth in *C. pepo*. (**A**) Germination rate of WT and *lox3a* mutant seeds under control (solid lines) and saline conditions (dashed lines). The seeds were imbibed for 16 h in distilled water (control) and 200 mM NaCl and then allowed to germinate between two filter papers soaked in the same solution in Petri dishes. The percentage of germination was assessed each 2 h up to 216 h. (**B**) WT and *lox3a* seeds at different hours after germination (HAG) under control and salt conditions. (**C**) Percentage of germination of WT and *lox3a* seeds at 48, 72, 96, and 120 h after imbibition in water and 200 mM NaCl. (**D**) Percentages of reduction in germination in response to salt stress in WT and *lox3a* seeds compared to those of the same genotype germinated in water. (**E**) Effect of *lox3a* mutation and salt stress on radicle length at 24 and 48 HAG. (**F**) Percentages of reduction in radicular length in response to salt stress in WT and *lox3a* seeds compared to those of the same genotype growing under control conditions. Data are mean ± SE (n = 70). The error bars represent SE. Different letters indicate statistically significant differences (*p* ≤ 0.05) between samples taken at the same time.

Figure 2 shows the germination rates of the WT and mutant *lox3a* under control and salt stress conditions for 216 h. We found no significant differences between the WT and *lox3a* at the onset of germination, and both the WT and mutant reached 50% of germination at 32 h (Figure 2A,B). However, *lox3a* reached a lower germination rate than the WT at 48, 96, and 120 h (Figure 2C). The final germination rate was significantly lower in *lox3a* (73.2%) than in the WT (88.7%) (Figure 2A), suggesting that JA is a positive regulator of germination in squash. NaCl treatment reduced germination rates over time in both the WT and *lox3a* with respect to control conditions, showing similar germination rates in the WT and mutant at all time points (Figure 2A,C). In fact, the effect of salt stress on germination, assessed as the percentage of reduction in the germination rate, was significantly the same in the mutant compared to the WT at all times evaluated (Figure 2D). 

Under control conditions, the radicle length of *lox3a* was differentially larger than that of the WT at 24 and 48 HAG (Figure 2B,E), indicating that JA inhibits radicle elongation. Salt stress reduced radicle elongation in both the WT and mutant (Figure 2B,E), with no significant differences in the percentage of reduction in radicle length between genotypes (Figure 2F). These data clearly showed that *CpLOX3A* (and JA) is required for both germination and inhibition of radicle elongation in squash, but it does not appear to be involved in the response to salt stress at this very early stage of plant development.

### 2.2. Crosstalk between ET, JA, and ABA during Germination under Normal and Salinity Conditions

**Figure 3:**
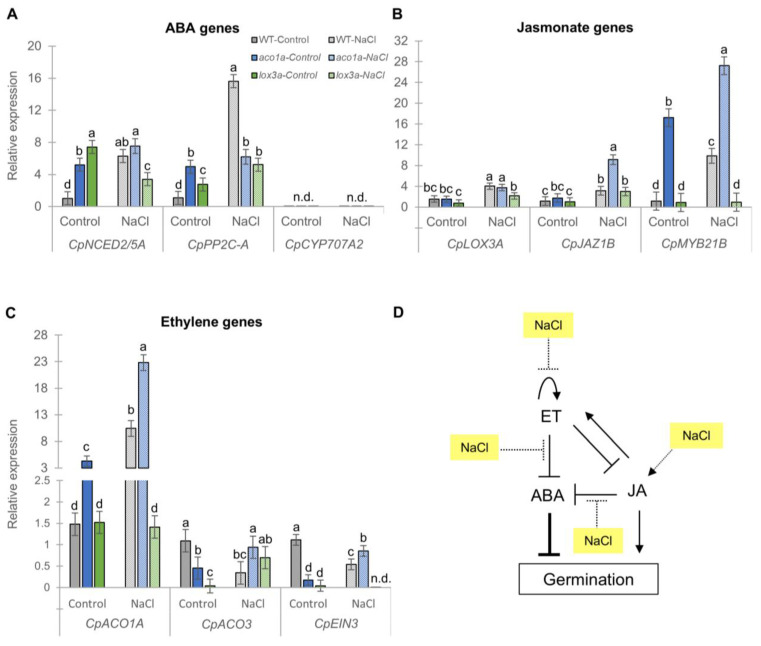
Effect of *aco1a* and *lox3a* mutations and salt stress on the relative expression of ABA, ET, and JA genes in imbibed seeds of *C. pepo.* (**A**–**C**) Expression profiles of ABA, JA, and ET genes in WT, *aco1a*, and *lox3a* seeds in response to salt. Seeds were incubated in 50 mL Falcon tubes containing 25 mL of distilled water (control) or 200 mM NaCl for 16 h at 24 °C in darkness under continuous shaking. The seeds detached from their coats were pulverized with liquid nitrogen, and total RNA was extracted. The relative level of each transcript was assessed by qRT-PCR in three independent replicates with at least 20 seeds per replicate and normalized by the 2^−ΔΔCT^ method. (**D**) Interaction model between ABA, JA, and ET in germination of *C. pepo* seeds under favorable conditions and in response to salt stress (dotted lines). Crosstalk between phytohormones is based on differential gene expression of WT vs. mutants *aco1a* and *lox3a* seeds soaked in water. The effect of NaCl on each hormone is based on differential gene expression of salt vs. control treatments in each genotype. n.d., not detected. The error bars represent SE. Different letters indicate statistically significant differences (*p* ≤ 0.05) between samples.

The crosstalk between ET, JA, and ABA in the regulation of germination was studied by comparing the transcription levels of different genes involved in the biosynthesis and signaling pathways of ABA, JA, and ET in the seeds of the WT, *aco1a*, and *lox3a* germinated under both water and salt conditions (Figure 3). 

The effects of *aco1a* and *lox3a* were determined by comparing gene expression in WT and mutant seed germinated in water. Under these conditions, the ABA biosynthesis and signaling genes *CpNCED2/5A* and *CpPP2C-A* were significantly more highly expressed in the mutants *aco1a* and *lox3a* than in the WT (Figure 3A), indicating that both ethylene and JA are negative regulators of ABA during germination (Figure 3D). No transcription of the ABA catabolism gene *CpCYP707A2* was detected in the seed of any of the genotypes upon germination (Figure 3A). The higher biosynthesis and signaling of ABA in *aco1a* and *lox3a* could explain their lower germination rates compared to the WT under control conditions. 

The effect of salt stress on the interactive model of ABA, ET, and JA was first assessed by comparing the expression of each gene under control and salinity conditions in the WT (Figure 3D). Then, it was determined whether those effects were similar or different in the *aco1a* and *lox3a* mutants, which means that the effect of salt was independent or dependent on ET or JA, respectively. Under salt stress, the reduced germination rate of WT seeds was accompanied by a prominent upregulation of the ABA genes *CpNCED2/5A* and *CpPP2C-A* in the WT (Figure 3A). However, this high induction of ABA biosynthesis and signaling genes was prevented in the *aco1a* and *lox3a* mutants (Figure 3A), suggesting that both ET and JA are required for ABA induction during squash seed germination under salinity (Figure 3D). This induction of ABA genes under salt stress was lower in *aco1a* and *lox3a* than in the WT (Figure 3A), which was accompanied by an increased germination rate in the ethylene mutant *aco1a*, but not in the JA mutant *lox3a*, suggesting that both ET and JA control germination through ABA, but JA also activates germination independently of ABA (Figure 3D).

Regarding the action of JA, we found that under control conditions the JA-deficient mutation *lox3a* did not alter the expression of any of the three JA genes tested (Figure 3B). However, the ethylene mutant *aco1a* greatly increased the expression of the JA signaling gene *CpMYB21B* (Figure 3B), indicating that ET negatively regulates the JA signaling pathway during germination (Figure 3D). Salt stress similarly increased the transcript levels of the three JA genes in the WT, *aco1a*, and *lox3a* seeds (Figure 3B), suggesting that NaCl modulates the JA pathway in an ET-independent manner (Figure 3D). The higher transcription levels of the JA genes in *aco1a* under salt stress could be due to the notably higher transcript levels found under control conditions compared to the WT (Figure 3B). Given that JA is a positive regulator of germination in squash, it is likely that JA mediated the increased salt tolerance of *aco1a* during germination. Furthermore, the larger radicle of *aco1a* in water, and the reduction in radicle elongation under salt stress, could also be mediated by the activation of the JA signaling pathway in the seed. 

Finally, in relation to ET genes, we found that *aco1a* and *lox3a* reduced the expression of *CpACO3* and *CpEIN3* under control conditions (Figure 3C), indicating positive feedback regulation of ethylene genes by ethylene, but also positive regulation of ethylene genes by JA (Figure 3D). The *CpACO1A* gene appears to be regulated differently, since *aco1a* was able to induce its expression compared to WT, but the *lox3a* mutation maintains the same expression as in WT (Figure 3C). Under salt stress, the ethylene genes did not respond in the same way either. *CpACO1A* was the only ET gene whose expression was highly upregulated in WT under salt stress compared to control conditions. In contrast, the expression ET biosynthesis and signaling genes *CpACO3* and *CpEIN3* were downregulated in response to salt in the WT (Figure 3C). When the *aco1a* seed germinated under salt stress, all ethylene genes were upregulated relative to what occurred in the WT, suggesting that the feedback regulation of ET genes was prevented under salt stress (Figure 3D). The mutation *lox3a* reduced the effect of salt on upregulation of *CpACO1A*, but enhanced downregulation of *CpEIN3*, which does not allow a clear conclusion to be drawn about the effect of salt on the interaction of JA and ET.

### 2.3. Response of aco1a and lox3a to Salt Stress during Seedling Etiolation in Darkness

Given that salt stress reduces the elongation of hypocotyl and roots during seedling growth [42], we set up a seedling etiolation assay in darkness that allowed us to compare the effect of salt stress on different genotypes. In this paper, we assessed the effect of salt stress on hypocotyl and root elongation and growth of *aco1a* and *lox3a* in comparison to the WT. 

**Figure 4:**
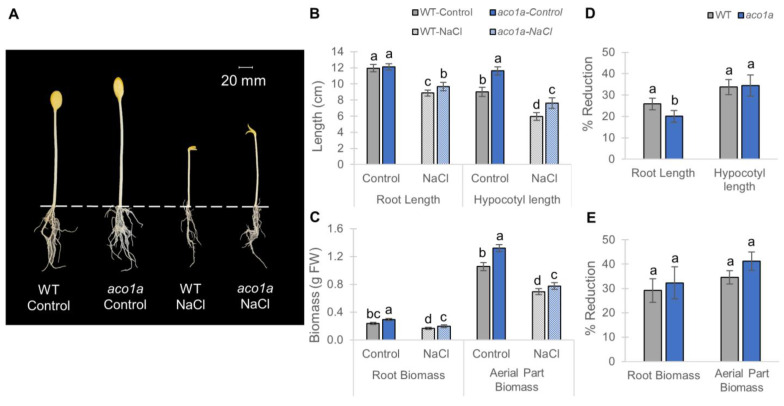
Response of *aco1a* to salt stress during seedling growth in darkness for 72 h. (**A**) WT and *aco1a* seedlings under control and NaCl treatments. (**B**) Effect of *aco1a* and salt stress on root and hypocotyl length. (**C**) Effect of *aco1a* and salt stress on the biomass of the root and aerial part. (**D**,**E**) Percentages of reduction in each growth parameter in response to salt stress in WT and mutant seedlings compared to those of the same genotype growing under control conditions. Dashed line separates roots from the aerial part. The error bars represent SE. Different letters indicate statistically significant differences (*p* ≤ 0.05) between samples.

Figure 4 shows the growth of the WT and *aco1a* seedlings after 72 h in darkness under control conditions and salt stress. Under standard conditions, the biomass of the root and aerial part, as well as the length of the hypocotyl, was significantly higher in *aco1a* than in the WT (Figure 4A–C), indicating that *aco1a* has higher vigor (understood as the rate of growth of the plant) than the WT and, therefore, that ET functions as an inhibitor of plant growth during seedling development. 

Hypocotyl and root growth parameters were reduced in response to NaCl treatment in WT and *aco1a* seedlings (Figure 4A–C), with no significant differences found between genotypes in the percentage of reduction in the parameters analyzed except that of the root length, which was significantly lower in the mutant than in the WT (Figure 4D,E). Hypocotyl length was reduced by approximately 34% in both genotypes; WT and *aco1a* root biomass exhibited a mean reduction of 29.16% and 32.31%, respectively; and the seedlings of the WT and *aco1a* reduced the aerial part biomass by a mean of 34.50% and 41.22%, but without significant differences between genotypes (Figure 4D,E). However, under salt stress, the root length of the WT seedlings was reduced by approximately 26%, while that of *aco1a* was reduced by only 20% (Figure 4D), indicating a higher salt tolerance of the root of *aco1a* during elongation. 

**Figure 5:**
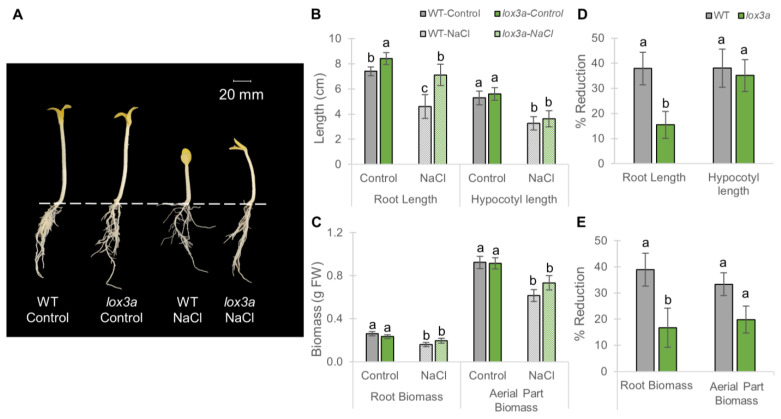
Response of *lox3a* to salt stress during seedling growth in darkness for 72 h. (**A**) WT and *lox3a* seedlings under control and NaCl treatments. (**B**) Effect of *lox3a* and salt stress on root and hypocotyl length. (**C**) Effect of *lox3a* and salt stress on the biomass of the root and aerial part. (**D**,**E**) Percentages of reduction in each growth parameter in response to salt stress in WT and mutant seedlings compared to those of the same genotype growing under control conditions. Dashed line separates roots from the aerial part. The error bars represent SE. Different letters indicate statistically significant differences (*p* ≤ 0.05) between samples.

Figure 5 compares the growth of *lox3a* and WT seedlings after 72 h in darkness under control and salinity conditions. We did not find significant differences in hypocotyl growth under control conditions between WT and *lox3a* seedlings (Figure 5A–C). However, the *lox3a* root was significantly larger than that of the WT (Figure 5B), suggesting that JA inhibits root elongation in the seedling stage. Salt treatment reduced all growth parameters in both the WT and mutant, but the root of *lox3a* was less sensitive to NaCl than that of the WT (Figure 5B–E). In fact, the root length of the WT was reduced by approximately 38%, while that of *lox3a* was reduced by only 15% (Figure 5D). Similarly, the WT root biomass exhibited a reduction of 39%, but that of the mutant decreased by only 17% (Figure 5E). In contrast, the aerial part was equally reduced in both WT and *lox3a* plants in response to salt (Figure 5D,E), suggesting that JA plays a more relevant role in the root than in the apical shoot in response to salinity.

### 2.4. Crosstalk between ET, JA, and ABA in the Root under Normal and Salinity Conditions

**Figure 6:**
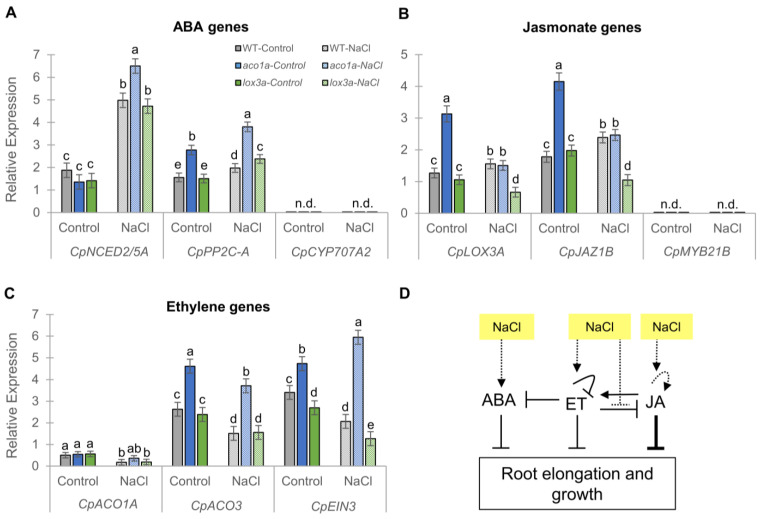
Effect of *aco1a* and *lox3a* mutations and salt stress on the relative expression of ABA, JA, and ET genes in the roots of squash seedlings. (**A**–**C**) Expression profiles of (**A**) ABA, (**B**) JA, and (**C**) ET genes on roots of the WT, *aco1a*, and *lox3a* in response to salt. The relative level of each transcript was evaluated by qRT-PCR in three independent replicates with at least 5–6 plants per replicate and normalized by the 2^−ΔΔCT^ method. (**D**) Interaction model between ABA, JA, and ET in *C. pepo* root under favorable conditions and in response to salt stress (dotted lines). The crosstalk between phytohormones is based on differential gene expression of WT vs. *aco1a* and *lox3a* roots under control conditions. The effect of NaCl on each hormone is based on the differential gene expression of salt vs. control treatments in each genotype. n.d., not detected. The error bars represent SE. Different letters indicate statistically significant differences (*p* ≤ 0.05) between samples.

As salt treatment did not affect hypocotyl growth, but only roots of mutants *aco1a* and *lox3a*, we focused our attention on studying the crosstalk between ET, JA, and ABA in seedling roots. To reach this goal, we compared the expression of ET, ABA, and JA biosynthesis and signaling genes in WT and mutant roots under control and salt stress conditions (Figure 6). This analysis could help to better understand the enhanced salt tolerance of both the *aco1a* and *lox3a* roots. Under normal conditions, the mutation *aco1a* had no effect on the expression of the ABA biosynthesis gene *CpNCED2/5A* compared to that of the WT, but the ABA signaling gene *CpPP2C-A* was found to be upregulated in the mutant compared to the WT (Figure 6A), indicating that ET inhibits ABA signaling during squash root development (Figure 6D). The *lox3a* mutation had no effect on the transcription of ABA genes (Figure 6A), which may indicate that the inhibitory action of ABA on squash root growth detected by Iglesias-Moya et al. [14] is likely not mediated by JA. In response to salt, the ABA genes were similarly upregulated in the root of the WT, *aco1a*, and *lox3a*, suggesting that NaCl modulates the ABA pathway in an ET- and JA-independent manner (Figure 6D). 

The JA biosynthesis and signaling genes *CpLOX3A* and *CpJAZ1B* were negatively regulated by ET in the root (Figure 6D). In fact, both JA genes were found to be upregulated in the *aco1a* root compared to the WT under control conditions (Figure 6B). However, no significant differences were found in JA gene expression between *lox3a* and the WT (Figure 6B). Salt stress increased the expression of *CpLOX3A* and *CpJAZ1B* genes in WT roots, but downregulated them in *aco1a* and *lox3a* roots (Figure 6B), meaning that JA induction in the root in response to salt is dependent on ET and JA (Figure 6D).

ET biosynthesis and signaling genes showed negative feedback regulation by ET in the root (Figure 6D). Therefore, under both control and salt conditions, the ET genes *CpACO3* and *CpEIN3* showed higher expression in the *aco1a* root compared to the WT (Figure 6C). The *lox3a* mutation led to a decrease in ET signaling compared to the WT (Figure 6C), suggesting that JA positively regulates ET during root development (Figure 6D). Salt treatment similarly reduced the expression of all analyzed ET genes in the roots of WT, *aco1a*, and *lox3a* plants, except that of the *CpEIN3* signaling gene in *aco1a* roots (Figure 6C), likely indicating that NaCl negatively regulates ET signaling in an ET-dependent and a JA-independent manner (Figure 6D).

Taking all the results into account, although the three phytohormones ABA, ET, and JA inhibit root elongation and growth, under salt stress this inhibition is mainly performed by JA. Thus, the WT root responded to salt stress by inducing ABA and JA and by repressing ET, which indicates that the action of JA and ABA in the inhibition of root growth under salt stress is higher than that of ET. Moreover, the salt tolerance of *aco1a* and *lox3a* roots was found to be associated with an upregulation of ABA and a downregulation of JA, which means JA prevails over ABA in the inhibition of root growth (Figure 6D). 

## 3. Discussion

This paper uncovers the crosstalk between ET, JA, and ABA in the regulation of germination and seedling establishment in *C. pepo* plants under both standard conditions and in response to salt stress. 

Contrary to the inhibitory effect of ABA on germination [14], the reduced germination rate of ET- and JA-deficient mutants *aco1a* and *lox3a* under standard conditions has demonstrated that ET and JA are positive regulators of squash germination. The phenotype of the ET-deficient mutant *aco1a* was similar to that found for the Arabidopsis ethylene-insensitive mutants *etr1* and *ein2* [43], and the squash ethylene-insensitive gain-of-function mutants *etr1a, etr1b*, and *etr2b* [31]. Previous reports showed that the function of ET receptors on germination does not follow the canonical ET signaling pathway, but appears to be mediated by ABA signaling in both Arabidopsis [44,45] and squash [14,31]. Therefore, it is likely that the function of *CpACO1A* and ET on germination is also mediated by the crosstalk with other phytohormones (see below). Similarly, the reduced germination of squash JA-deficient mutant *lox3a*, as previously reported for JA biosynthesis mutants in Arabidopsis [46,47,48,49,50,51], supports a positive role of JA in squash germination. However, JA should also crosstalk with other phytohormones signaling pathways, since mutations in JA signaling genes such as *COI1*, *JAZs*, and *MYC2* do not lead to impaired germination phenotypes under standard conditions in Arabidopsis [52,53,54,55,56].

Our gene expression data in WT and mutants have shown that the positive action of ET and JA in germination is mediated by the downregulation of ABA biosynthesis and signaling pathways. This crosstalk between ET and ABA was also shown in Arabidopsis on the basis of ethylene-insensitive mutants *etr1* and *ein2* [43] and *etr1-2* [57]. Moreover, the reduced ABA content and ABA insensitivity of the squash ethylene-insensitive mutant *etr2b* also indicated that ABA mediates the action of ethylene receptor CpETR2B in squash seed upon germination [14]. JA and ABA mutants of Arabidopsis have also confirmed a crosstalk between JA and ABA in germination [52,53,58,59,60,61,62,63,64,65,66]. ABA treatment also affected the germination rate of some JA mutants [53,54,55,56,64], which indicated that JA regulates seed germination by modulating the ABA signaling pathway. 

Little is known about the crosstalk between ET and JA during germination. Pluskota et al. [67] reported that transcript levels of *SlNP24*, encoding an osmotin-like protein, and the upstream ethylene transcription factor gene *TERF1*, increased in response to MeJA during tomato germination, which supports our results indicating that JA positively regulates ET biosynthesis and signaling genes such as *CpACO3A* and *CpEIN3* during seed germination. Therefore, JA not only modulates the germination of squash by downregulating ABA, but also by upregulating the biosynthesis and the action of ET. On the other hand, after assessing the level of different phytohormones in Arabidopsis *etr1-2* seeds, Chiwocha et al. [57] found that ET can regulate phytohormones such as ABA, auxin, indole-3-aspartate, cytokinin glycosides, and gibberellins during germination. Similarly, the upregulation of JA genes in the ET-deficient mutant *aco1a* of squash also indicates that the action of ET in squash germination depends on the regulation of both ABA and JA (Figure 3). 

Salinity and other adverse environmental conditions reduce seed germination, which suppresses the germination and growth of plants until favorable environmental conditions are reestablished. The comparison of germination in WT, *aco1a*, and *lox3a* seeds under control and NaCl treatments defined those pathways where salt stress interacts with ABA, ET, and JA (Figure 3), and demonstrated that ET is a negative regulator of salt tolerance in squash, but JA does not seem to mediate the response to salt stress during germination. Salt treatment upregulated the transcription of JA genes during squash germination, which agrees with previous research in other species [68,69,70,71,72]. The dramatic induction of *LOX3* by salt in Arabidopsis and the increased sensitivity of the *lox3* mutant to salt compared to the WT during germination indicate that *LOX3* and JA play a positive role in Arabidopsis response to salt stress [73]. However, squash *lox3a* and WT seeds showed the same germination rate under salt stress, suggesting that other LOX enzymes are able to complement the loss of function of CpLOX3A.

The enhanced salt tolerance of the *aco1a* mutant during germination indicates that ET plays a negative role in salt tolerance during squash germination, similarly to what occurs in rice and tobacco [26,30]. In accordance with previous results in squash ET-insensitive mutants [14,31], we found that the *aco1a* mutation led to a lower NaCl-mediated induction of ABA genes in the mutant compared to the WT, demonstrating that the inhibitory action of ET in seed germination under salt stress appears to take place by the mediation of ABA signaling and not by the canonical ET signaling pathway, as also found in Arabidopsis [44,45]. Furthermore, the gain-of-function *etr* mutants of *C. pepo*, including *etr1a*, *etr1b*, and *etr2b*, are all more tolerant to salt stress during germination [31], which was found to be also correlated with a reduced ABA content and a reduced expression of ABA biosynthesis and signaling genes during germination [14]. 

The ET-deficient mutation *aco1a* promoted plant growth in squash, resulting in longer radicles during early root elongation and seedlings with higher height and biomass of both leaves and roots. Mutations in ET receptor genes in *C. pepo* confer ethylene insensitivity and were also found to be more vigorous [31], at both seedling and adult stages of the plant. Therefore, ET has been shown to be a negative regulator of plant growth throughout the entire plant life cycle. The same phenotype was found in Arabidopsis ethylene-insensitive gain-of-function mutants [74,75,76], and in the ET-deficient loss-of-function mutant *acs7* [77]. 

The *aco1a* seedlings were also more vigorous than those of the WT under salt treatment, indicating that the *aco1a* mutation confers an adaptive advantage for the salt stress response, and therefore that ET negatively regulates the salt tolerance response in squash. The higher growth rate and vigor of ET-insensitive squash mutants in response to salt stress [31] also support this conclusion. In this paper we have also demonstrated that JA mediated the enhanced salt tolerance of the *aco1a* root during seedling establishment. Although both the ABA and JA genes were induced in the WT root in response to salt, the *aco1a* mutation led to greater transcription levels of the ABA genes, but to a repression of the JA genes. Given that both ABA [14] and JA (see below) inhibit root elongation and growth in squash, the higher tolerance of *aco1a* roots to salt suggests that JA plays a more relevant role than ABA in the inhibition of root growth under salt stress. Moons et al. [32] also reported that ABA had a smaller inhibitory effect on primary root elongation than JA in rice seedlings. However, ABA not only inhibits root growth, but also positively regulates salt tolerance, limiting water loss in leaves [78,79], and inducing the expression of a large set of genes that are involved in membrane protection [80], ion homoeostasis, and osmotic adjustment [81]. In fact, enhanced salt tolerance of *etr2b* plants was found to correlate with a greater induction of ABA biosynthesis and the intracellular Ca^2+^ signaling pathway [31]. Therefore, we cannot exclude that the enhanced salt tolerance of the *aco1a* roots is mediated not only by JA but also by ABA.

However, although the *lox3a* mutation had no effect on the vigor of the aerial part of the plant, the mutant showed longer roots compared to WT, both during the early elongation of the roots and the seedling stage. This demonstrates that JA inhibits primary root elongation under standard and stress conditions in *C. pepo*, which is consistent with what occurs in other plant species [82,83,84,85]. Moons et al. [32] also reported that the effect of JA on the inhibition of the rice shoot growth was less pronounced than the effect of ABA. In the root, however, the percentages of reduction in both length and biomass in response to salt were significantly lower in the *lox3a* mutant than in the WT, suggesting that *CpLOX3A* and JA are negative regulators of salt tolerance during root development in *C. pepo*. This conclusion contrasts with what has been found in *Arabidopsis thaliana*, *Pisum sativum* (pea), *Hordeum vulgare* (barley), *Triticum aestivum* (wheat), and *Glycine max* (soybean) [33,34,35,36,73], but agrees with data in other crops. Thus, the root of the JA biosynthesis mutants *cpm2* and *hebiba* of rice were found to be more tolerant to salt than that of the WT [86]. Likewise, the overexpression of the Cytochrome P450 family gene *CYP94C2b* (*CYP94*), encoding an enzyme that inactivates the bioactive form of JA, JA-Ile (JA–isoleucine), improved the viability of rice plants under saline conditions [39]. Furthermore, overexpression of *JAZ9* (jasmonate ZIM domain 9) in rice alleviated growth inhibition caused by salt and water stress [37]. The Arabidopsis JA response mutants, such as *coi1* and *jin1*, also exhibited tolerance to moderate drought stress [87]. Finally, in a comparison of two grapevine cell lines that differed in their salinity tolerance, the accumulation of JA and JA-Ile was more pronounced in the sensitive *Vitis riparia* rather than in the salt-tolerant *Vitis rupestris* [38]. 

Compared to the WT, the roots of the mutant *lox3a* showed higher induction of ABA signaling but repression of JA genes under salt stress. These results again support the hypothesis that JA plays a more relevant role than ABA in inhibiting root elongation under salt conditions. However, the enhanced salt tolerance of *lox3a* could be mediated by other phytohormones such as ABA. In Arabidopsis there is a negative interaction between ABA and JA during the response to salt stress, which occurs through the MYC2 transcription factor and the *JASMONATE ZIM-DOMAIN* (*JAZ*) gene family [88].

Finally, salt tolerance phenotypes were found in roots of both *aco1a* and *lox3a* mutants during seedling establishment, but not during early radicle growth, probably due to a concentration effect. We found that 200 mM NaCl was an optimal concentration to assess seed germination and differentiate between salt-sensitive and salt-tolerant genotypes during this stage, but this concentration of NaCl inhibited radicle elongation too much. However, the concentration used at seedling stage, 100 mM NaCl, allowed the discovery of differences in both apical shoot and root growth between genotypes.

## 4. Materials and Methods

### 4.1. Plant Material

In this study, we analyzed the response to salt stress of two mutants that were deficient in ET (mutant *aco1a*) or JA (mutant *lox3a*). Both mutants were previously identified by their floral phenotype from a *Cucurbita pepo* EMS mutant collection from the Vegetable Genetics and Breeding group of the University of Almeria (Spain) [40,41]. The *CpACO1A* gene encodes a type I ACO enzyme involved in ethylene biosynthesis, while *CpLOX3A* encodes a lypoxygenase gene involved in JA biosynthesis. The mutants were maintained in BC_2_S_1_ segregating generations, obtained after crossing each mutant twice with the background genotype MUCU16, and then selfed.

### 4.2. Seed Germination under Water and Salt Stress

Seed germination of *aco1a* and *lox3a* BC_2_S_1_ segregating populations was tested under control and salt stress conditions. Seeds were incubated in 50 mL Falcon tubes containing 25 mL of distilled water (control) or 200 mM NaCl for 16 h at 24 °C in darkness under continuous shaking. After imbibition, seeds were sown in Petri dishes between two filter papers moistened with the corresponding solution. The Petri dishes with seeds were then incubated in a growth chamber in darkness at 24 °C and 80% relative humidity (RH) for 9 days. Quantities of 550 *aco1a* and 550 *lox3a* BC_2_S_1_ seeds (≈70 homozygous mutant seeds and ≈70 homozygous WT seeds per treatment) were germinated and grown in distilled water and saline solution in two independent experiments. Digital images of the germinated seeds were recorded 24 and 48 h after germination (HAG), and were processed using ImageJ^®^ v1.52a to evaluate radicle elongation of both WT and mutants. The seeds were considered germinated when the seed coat was broken and the primary root protrusion was visible (>1 mm). Separation of WT and mutant seeds in BC_2_S_1_ segregating populations was carried out by genotyping for causal mutations of the phenotypes using individual DNA from radicles. The concentration of NaCl used for this assay was selected after analyzing the effect of different concentrations of NaCl (85, 150, 200, 250, and 300 mM) on the seed germination of the genetic background of the mutant collection MUCU16, as previously described in Alonso et al. [89].

### 4.3. Seedling Etiolation under Salt Stress

Seedling etiolation of *aco1a* and *lox3a* BC_2_S_1_ segregating populations was evaluated in seedlings grown under 100 mM NaCl in two independent experiments. A total of 240 seeds of the BC_2_S_1_ segregating population of *aco1a* or *lox3a* (≈30 homozygous mutant plants and ≈30 homozygous WT plants per treatment) were imbibed in distilled water under the same conditions described in the previous section and then incubated in Petri dishes for 72 h. After this time, each seed was sown in pots containing vermiculite and irrigated with a control or salt solution. The control and saline solutions were prepared with distilled water supplemented with nutrient solution (1 g/L) and the pH was adjusted to 5.8. A quantity of 100 mM NaCl was added to the saline solution. The conductivities of the control and salt solutions were 1.725 and 11.573 dS/m, respectively. Pots with germinated seeds were incubated in the growth chamber in darkness under the same conditions described for germination. After 72 h, growth parameters (biomass and length of roots and aerial part) were measured in etiolated seedlings after extracting them from the pot and washing them to remove vermiculite. Furthermore, root samples were collected and immediately stored on dry ice to assess the expression of different genes. Separation of WT and mutant seedlings was performed after genotyping for *aco1a* or *lox3a* mutations using individual DNA from cotyledons. The concentration of NaCl used for this assay was selected after analyzing the effect of different concentrations of NaCl (30, 60, 100, and 150 mM) on seedling growth of the genetic background of the mutant collection MUCU16, as previously described in Alonso et al. [89].

### 4.4. SNP Genotyping for Validating aco1a and lox3a Mutations in BC_2_S_1_ Segregating Populations

Since BC_2_S_1_ segregating populations were used in all assays, subsequent identification of seed and seedling genotypes was necessary. Homozygous WT and mutant seeds/plants were identified by detecting WT and *aco1*a or *lox3a* alleles using Kompetitive allele-specific PCR technology (KASP). DNA was isolated from the radicle of germinated seeds or cotyledons of etiolated plants following the CTAB protocol [90]. Primers were synthesized by LGC Genomics^®^ and the LGC protocol was followed. Multiplex PCRs were performed in the FX96 Touch Real-Time PCR Detection System (BioRad^®^, Hercules, California, USA) in a final reaction volume of 10 μL, containing 5 μL KASP V4.0 2x Master mix (LGC Genomics^®^), 0.14 μL KASP-by-Design primer mix (LGC Genomics^®^), 2 μL of genomic DNA 10–20 ng/μL genomic DNA, and 2.86 μL of water. The data were analyzed, and SNP genotypes were identified using CFX Maestro^TM^ software, v2.3 (Bio-Rad^®^).

### 4.5. Assessment of Relative Gene Expression by Quantitative RT-PCR

The transcription of genes involved in the ABA, ET, and JA pathways was assessed by quantitative reverse transcription PCR (qRT-PCR). Table S1 shows the genes and sequences of the primers used for each qRT-PCR reaction. *CpNCED2/5A* and *CpPP2C-A* are genes for ABA biosynthesis and signaling, respectively, while *CpCYP707A2* is involved in ABA catabolism. The *CpACO3* and *CpACO1A* genes are involved in ET biosynthesis, while *CpEIN3* is a positive regulator of ethylene signaling. *CpLOX3A, CpJAZ1B*, and *CpMYB21B* are JA biosynthesis and signaling genes, respectively. Samples for gene expression analysis included seeds without a seed coat soaked in distilled water and 200 mM NaCl for 16 h, and roots of etiolated seedlings in darkness for 72 h under control and saline conditions (100 mM NaCl). The analysis was carried out in three biological replicates per genotype and treatment, each composed of a set of 20 seeds or 5–6 different plants of the same genotype and 3 technical replicates. The plant material was collected and pulverized in liquid nitrogen, and total RNA was isolated according to the GeneJET Plant RNA Purification Kit (Thermo Fisher^®^) and converted to cDNA with the cDNA RevertAid^TM^ Kit (Thermo Fisher Scientific^®^). qRT-PCR was performed on a 96-well plate using the thermocycler of the CFX96 Touch Real-Time PCR Detection System (Bio-Rad^®^), in 10 µL total volume of 10 L with SYBR Green PCR Master Mix (Bio-Rad^®^). Quantities of 5 μL of Green qPCR Super Mix (BioRad^®^), 0.5 μL each of the forward and reverse primers (10 μM), 2 μL of DEPC-water, and 2 μL of cDNA diluted at 1:4 formed each qPCR reaction. The 2^−ΔΔCT^ method was used to calculate gene expression values [91]. The WT gene expression values were calculated as the average of the relative expression values of the WT coming from the segregating populations of *aco1a* and *lox3a*. The constitutive *CpEF1α* gene was used as a reference to normalize the gene expression results. 

### 4.6. Statistical Analyses

Data were subjected to a one-way analysis of variance (ANOVA) using the statistical software Statgraphic Centurion XVIII v18.1.16. Differences between genotypes and treatments were separated by the least significant difference (LSD) at a significance level of *p* ≤ 0.05.

## Data Availability

All relevant data can be found within the manuscript and its Supplementary Materials.

## Supplementary Materials

The following supporting information can be downloaded at: https://www.mdpi.com/article/10.3390/ijms25168728/s1.
