# Supplementary material for: Crosstalk between Ethylene, Jasmonate and ABA in Response to Salt Stress during Germination and Early Plant Growth in Cucurbita pepo
Source: Int J Mol Sci. 2024 Aug 10;25(16):8728. doi: 10.3390/ijms25168728 (PMC11354493; doi:10.3390/ijms25168728)
Supplement: Supplementary file 1 [file ijms-25-08728-s001.zip › ijms-3119614-supplementary.pdf]

Supplementary Table S1. List of primer sequences used for qRT-PCR analysis.

Gene	Gene ID (Cucurbit Genetic Database)	Gene Pathway	Forward Primer	Reverse Primer
CpNCED2/5A	Cp4.1LG10g05900	ABA biosynthesis pathway	TCTGGCTCAAGAACAACAGC	GTGGTACGGCAAATCGTCTT
CpPP2C-A	Cp4.1LG03g14520	ABA signaling pathway	TCAGAGCTGCACGCCGC	GAATCTTTGCAAAACAAGGAACAG
CpCYP707A2	Cp4.1LG02g13560	ABA catabolism pathways	CGGCGACAAAGTGGAGAAAA	CGAACTCCACCTCTTCCACT
CpACO1A	Cp4.1LG04g02610	ET biosynthesis pathway	CATAGAGTGATGACTCAACAAGC	CCATTGGACCCAAATTAGCA
CpACO3	Cp4.1LG10g09730	ET biosynthesis pathway	GGAGGGAGAGGAAGATAAGG	TGGGTTATTGGAAAATGGAG
CpEIN3	Cp4.1LG18g01370	ET signaling pathway	TAGCAGCCAATTCAACCAGTTTAAGCC	CGGTAAAGCATCGAATTGAGATCAGG
CpLOX3A	Cp4.1LG12g09270	JA biosynthesis pathway	AACGCGCACAATCTTCTTCT	AGCTGCCATATCCAATTGCT
CpJAZ1B	Cp4.1LG17g04050	JA signaling pathway	CCATACCAAATGAACCAGCAG	GGGGATAGATATGAAAAACCACG
CpMYB21B	Cp4.1LG04g05410	JA signaling pathway	ACTCAGCCGAAGGTGTGGT	ATGTTGCCTCGTCGAACATT
CpEF1α	Cp4.1LG12g00880	Reference gene	CGTCAAGAAGAAATAAGCCA	CTACTACGAGAGAGAGAGCCG
